# Challenges and Practices in the Analysis of Silicon Kerf from the PV Industry by Combinatorial Analytical Methods

**DOI:** 10.3390/ma19030541

**Published:** 2026-01-29

**Authors:** Tinotenda Mubaiwa, Marisa Di Sabatino, Sergey Khromov, Marthe Nybrodahl, Alexander Azarov, Jafar Safarian

**Affiliations:** 1 Department of Materials Science and Engineering, Norwegian University of Science and Technology, N-7491 Trondheim, Norwayjafar.safarian@ntnu.no (J.S.); 2 SINTEF AS, SINTEF Industry, Metal Production and Processing, P.O. Box 4760, N-7465 Trondheim, Norway; marthe.nybrodahl@sintef.no; 3 Department of Physics, Centre for Materials Science and Nanotechnology, University of Oslo, P.O. Box 1048 Blindern, N-0316 Oslo, Norway

**Keywords:** GDMS, SIMS, kerf, silicon, LECO, recycling

## Abstract

Exploitation of waste streams has gained prominence not only in sustainable use of resources but also as a potential source of raw materials. Silicon kerf is one such waste stream and its recycling has been quite topical in recent years. In the present study, the characterization of different industrial kerf samples was carried out using several techniques. The average metallic impurity concentration was approximately 400 ppmw with average particle size (D50) of 3.5 µm and surface area of approximately 33 m^2^/g. The inhomogeneity of kerf was shown to pose challenges like potential isotope interferences during analysis as well as being susceptible to high uncertainties and relative standard deviation (RSD). Remedies and best practices were recommended for successful characterization of such inhomogeneous materials.

## 1. Introduction

The performance of photovoltaic (PV) solar materials is dependent on the amount of impurity elements. Any slight deviation from the threshold quantities can have detrimental effects on performance and quality, especially with the ever-increasing stringent quality requirements on PV solar materials [1,2]. It is thus critical to have close control over impurities for such applications. The best way of monitoring and performing quality control is by tracking impurity concentration during and after processing. This is usually done by using analytical techniques such as glow discharge mass spectrometry (GDMS) [3,4,5], inductively coupled plasma mass spectrometry (ICP-MS) [6,7], and secondary ion mass spectrometry (SIMS) [8,9] among others. Impurity elements, especially transition metals as low as a few ppmw can reduce solar cell efficiency quite significantly.

Silicon kerf (sawdust) is produced during the diamond wire sawing process for solar cell wafers and its recycling into PV solar or related applications has gained increasing attention. Some of the applications include anode materials for battery applications [10], thermoelectrics, ceramics [11], PV feedstock and metallurgical applications [12], cement, glass [13] or hydrogen applications, just to mention a few. It should be noted that most of these applications are under research and development, and there are not many commercial applications at present.

Søiland et al. [14] recently characterized and tested the performance of silicon kerf powder as a potential anode material for Li-ion batteries and concluded that silicon kerf can indeed be used for such applications as it possesses similar properties to nano-sized Si. Zheng et al. also recycled silicon kerf by hydrothermal processes to produce anode material, again for lithium-ion batteries [15]. Mesaritis et al. [16] also prepared thermoelectric magnesium silicides from recycled silicon kerf, evaluated their performance and concluded that the silicides have great potential for application as thermoelectrics. SiC ceramics were produced from Si-kerf by Ren et al. [17], where they tested the effect of sintering conditions on the properties of the ceramics. These are some of the recent applications for recycled Si-kerf and more applications are still being developed.

In this context, the silicon kerf recycling back into the PV value chain or other applications is dependent on accurate measurement of impurity content.

GDMS has established itself as the preferred technique for trace elemental impurity analysis in the semiconductor industry. This is due to its relatively short analysis time and easy sample preparation, low detection limits and reasonably low cost [18,19]. It has been shown that GDMS can also be applied to powder samples and that relative sensitivity factors for bulk solid samples can be applied to powder samples [20,21]. It is worth noting that when analyzing silicon powder samples by GDMS, the results are more sensitive to the homogeneity of the powder compared to bulk analysis. Furthermore, the GDMS method cannot measure elements such as H, C, N and O [22]. The main causes for this are the polyatomic interferences from the argon plasma, their low ionization/sputtering yields, and the challenges in finding calibration standards [23].

The SIMS technique is very good for surface analysis with a wider elemental range and sensitivity [22,24]. The factors responsible for this include how secondary ions are generated and detected at the surface, the flexibility of primary ions as well as the ability to detect both elemental and molecular ions. However, SIMS has its own challenges when it comes to bulk analysis of samples. Inhomogeneity in bulk materials is highly magnified, which can be problematic, especially for silicon kerf samples which are quite inhomogeneous. Also, the analysis time of SIMS is relatively longer compared to GDMS [25]. Carbon and oxygen are important impurities in solar PV applications which have adverse effects if threshold limits are exceeded. Thus, the importance of their accurate measurement and detection cannot be overemphasized. One well-established method of measuring such elements is the inert gas analysis (IGA) and infrared (IR)-combustion method which can analyze volatile elements such as C, O, H, N and S [26,27,28], based on combustion or inert gas fusion as mentioned earlier.

Although characterization is central to the recycling and performance of silicon kerf and related products, there is not much literature available with extensive characterization of different kerf samples from different sources. To the best of our knowledge there currently is no study aimed at not only extensively characterizing different silicon kerf samples but also focusing on the characterization techniques themselves, shedding light on common challenges encountered during characterization of inhomogeneous silicon kerf samples. In the present study, different industrial silicon kerf samples from different sources are characterized using GDMS, SIMS, IGA and IR combustion methods. The differences and similarities in chemical and structural properties of different industrial silicon kerf samples are outlined, providing useful information for standardized treatment and potential recycling. We also highlight the common challenges encountered in powder and bulk solid silicon kerf samples such as potential isotope interferences and high measurement uncertainties due to inhomogeneity and how they can be overcome from a practical viewpoint.

## 2. Experimental Procedure, Instrumentation and Methodology

### 2.1. Materials

Solar cell silicon is a high-purity material which requires very sensitive analytical methods for quantification of different elements. In the present study, several characterization techniques were employed, namely scanning electron microscopy/energy dispersive X-ray spectroscopy (SEM/EDS), X-ray diffraction (XRD), physisorption of inert gas by the Brunauer–Emmett–Teller (BET) method, infrared absorption and thermal conductivity to measure combustion gases within the kerf sample using the IGA and IR combustion methods, particle size distribution (PSD) analysis and glow discharge mass spectrometry (GDMS).

#### 2.1.1. Sample Storage and Handling

Five (5) industrial silicon kerf samples, namely REC1, REC2, REC3, REC4 and REC5 from a local company but sourced from different suppliers (mostly from China) were investigated, aiming to compare the similarities and differences in their properties, as well as suggesting standardized treatment methods. The samples were shipped in cake form, in sealed metal containers. They were kept and stored in these containers for several months before characterization. Firstly, the samples were dried in an oven at 80 °C for 24 h to remove moisture and then crushed using ceramic mortar and pestle to produce silicon kerf powder. The powders were then taken for analysis using different techniques.

#### 2.1.2. Morphology, Particle Size and Surface Area

The as-received dried and crushed silicon kerf samples were analyzed by SEM Zeiss ULTRA 55(Carl Zeiss Microscopy GmbH, Oberkochen, Germany), a scanning electron microscope (SEM) with a field emission electron gun (FEG), as well as the JEOL-IT800-FEG-SEM (JEOL Ltd., Tokyo, Japan). XRD analysis was done by a routine powder X-ray diffractometer (Bruker D8 ADVANCE DaVinci (Bruker Corp., Bremen, Germany)) with CuKα radiation. Particle size distribution was analyzed by a Horiba-Partica LA-960-Particle Size Distribution Analyzer(HORIBA Ltd., Kyoto, Japan), while BET surface area was analyzed by Micromeritics 3Flex 3500(Micromeritics Instrument Corp., Norcross, GA, USA).

#### 2.1.3. Elemental and Bulk Chemical Analysis

Powder silicon kerf was analyzed by GDMS as well as the IGA and IR combustion methods while solid silicon from remelting of kerf was analyzed by SIMS. Some powder samples were also sent to an external laboratory for inductively coupled plasma optical emission spectrometry/mass spectrometry (ICP-OES/MS) analysis. For analysis of solid samples, 10 mm × 5 mm samples were prepared for SIMS.

### 2.2. Glow Discharge Mass Spectrometry (GDMS)

#### 2.2.1. Instrumentation and Methodology

This study was carried out using an Astrum instrument (Nu Instruments, Ametek Inc., Wrexham, UK), a double-focusing, low-flow dc-GDMS, similar to the VG 9000 [29,30]. The experiments were performed using a flat cell configuration. The front anode plate had a 10 mm opening.

The GDMS instrument was tuned and calibrated across a range of masses using tantalum. The calibration included the following masses: ^12^C^+^, ^36^Ar^+^, (^40^Ar)^2+^, (^40^Ar)^3+^, ^181^Ta^+^, and ^181^Ta^+40^Ar^+^, under discharge conditions of 2 mA and 1200 V. A ^181^Ta signal intensity of 2 × 10^−9^ A was recorded during a magnet scan at a mass resolution of approximately 4000 (M/ΔM, 10% of peak height). The glow discharge cell was cooled with liquid nitrogen, and argon gas of 99.9999% purity was used as the discharge gas.

In GDMS, the influence of different sample matrices and discharge conditions on quantification is generally considered to be minor [31]. Discharge, matrix and element-specific effects are accounted for in the analysis using the so-called relative sensitivity factors (RSFs) [31,32]. This relationship is expressed in Equation (1), where C_X/M_ represents the concentration of element X in sample matrix M, and I_X_ and I_M_ denote the respective measured ion beam intensities, corrected for abundance.

(1) $$C_{X/M}=\frac{RSF_{X}}{RSF_{M}}\times \frac{I_{X}}{I_{M}}$$

All reported concentrations were determined using the standard RSFs supplied with the instrument, which are based on the RSFs provided by Vieth and Huneke [30].

#### 2.2.2. Sample Preparation for GDMS

For GDMS analysis, solid silicon samples were cut using a diamond blade, mechanically polished to a 1200 SiC grit finish, rinsed with ethanol, and air dried. Powder samples cannot be analyzed directly by GDMS. A commonly used method, which we utilized, involves pressing the powder into a secondary cathode made of a high-purity metal, typically indium [33,34]. During plasma ignition, both the powder and the secondary cathode are co-sputtered, enabling the elemental analysis of the powder material.

Indium secondary cathodes were prepared using high-purity 7N indium pellets sourced from RASA Industries Ltd., (Tokyo, Japan). The pellets were flattened to a thickness of 0.2 mm by pressing them between two polytetrafluoroethylene (PTFE) sheets [35]. The PTFE sheets serve to maintain a clean and smooth surface, preventing contamination or unwanted surface structuring during the pressing process. Pressing was performed using a manual RS Pro arbor press with a maximum output force of 1 ton. To eliminate surface contaminants and any oxide layers, the masks were etched in concentrated 65% nitric acid for 1 min. After etching, they were rinsed with deionized water and ethanol, followed by air drying. Due to the high purity of the indium used as the cathode material, its contribution to the analytical results can be considered negligible (Table 1). Si-kerf powder was embedded into the pellets by gentle manual pressing. Rubber gloves were worn, and plastic bags were used to preserve sample integrity.

Another potential source of contamination is the Al_2_O_3_ insulator plates within the sample holder assembly. However, previous measurements have shown that they contribute only approximately 30 ppb of aluminum, which is also considered negligible for the purposes of this study [36].

### 2.3. Secondary Ion Mass Spectrometry (SIMS)

SIMS analysis was performed using CAMECA 7F microanalyzer(CAMECA, Janvilliers, France) at University of Oslo. The instrument is equipped with two ion sources, i.e., duoplasmatron and Cs ion source. Duoplasmatron is used to extract O_2_^+^ primary beam, which is typically used to enhance ionization efficiency of electropositive elements (the left part of the Periodic Table), while Cs ion source is intended to analyze the electronegative elements (the right part of the Periodic Table). The instrument can operate in three different modes: depth profiling, mass spectrum, and imaging for a detection of practically any element of the Periodic Table. Similarly to GDMS, a high mass resolution can be used to avoid mass interference and enhance the detection efficiency of some elements. Prior to measurements, the samples were polished to get a flat surface since any surface roughness can negatively affect the accuracy of the results.

In the present work the depth profile measurements were performed with the 10 keV O_2_^+^ or 15 keV Cs^+^ primary beam rastered over the 150 × 150 µm^2^ area and only the central part of the crater was used to collect the SIMS signal. The SIMS intensity-to-concentration calibration was performed using ion-implanted samples since implantation is a convenient technique providing a controllable introduction of an element of interest to the specific depth with the concentration not limited by a solid solubility. In its turn, the time-depth conversion has been performed by measuring the sputtered crater depth using a Dektak 8 stylus profilometer and assuming a constant erosion rate. The sputtering rates were 81 and 132 nm/min for the measurements performed with O_2_ and Cs ions, respectively.

### 2.4. Inert Gas Analysis (IGA) and Infrared (IR)-Combustion Methods

IGA was performed using the LECO ON836 (LECO Corporation, Täby, Sweden) apparatus in order to determine the oxygen and nitrogen concentration of the silicon kerf samples. IR-combustion analysis was used to determine the carbon concentration and was performed using the LECO CS844 (LECO Corporation, Täby, Sweden). LECO CS844 can also be used to measure the sulfur content, but this was not done in this study. Instrument ranges and precisions for the two instruments are listed in Table 2 [37]. Both instruments use the LECO Cornerstone software (LECO Cornerstone ON836 Version 2.4.5 and LECO Cornerstone CS844 Version 2.5.5) for analysis control and result treatment.

These instruments allow for easy and accurate determination of the elemental composition of the samples. An additional benefit is that the analysis only requires small sample amounts, though the process is destructive. Before analyzing the samples, a series of blank samples is measured to establish a baseline. Then, standards of known oxygen or carbon contents are used to calibrate the instrument. For this study, steel samples were used as standard. The elemental concentration of the silicon kerf samples is determined relative to the calibrated steel standard. Both powder and larger bulk specimens can be analyzed. At least three parallels were analyzed for each sample. To prevent contamination, all specimens and other relevant components (i.e., standards, crucibles) are handled using gloves and clean tools.

#### 2.4.1. Theory of Operation—LECO ON836

The LECO ON836 is ideal for the elemental analysis of inorganic materials, ferrous and non-ferrous alloys and refractory materials [37]. To analyze the oxygen and nitrogen content, the sample is weighed out in a nickel capsule, which is then added to a small graphite crucible. The crucible is then moved into the impulse furnace, where the graphite crucible is heated by passing a current through it under an inert helium atmosphere. The oxygen in the sample reacts with the carbon from the graphite crucible to form carbon monoxide (CO) and carbon dioxide (CO_2_). The helium carrier gas sweeps the analyte gases out of the furnace and through a mass flow controller, where CO and CO_2_ are detected by non-dispersive infrared (NDIR) cells. The cells work by separating gases based on their different absorption of IR-energy at different wavelengths in the IR-spectrum. The nitrogen in the sample is released as molecular nitrogen (N_2_) and is detected using the difference in thermal conductivity (TC) between the carrier and analyte gas [38,39].

#### 2.4.2. Theory of Operation—LECO CS844

The LECO CS844 is ideal for a wide range of materials such as steels, ores, ceramics and other inorganic materials [39]. To analyze the carbon and sulfur content, the sample is weighed out and added to a small ceramic crucible. In addition to the sample, an accelerator is added to speed-up the combustion reaction by igniting the sample. It also doubles as an oxide-dissolving flux to promote better fluidity. In this study, copper chips and iron chips were used as accelerators. The sample then gets moved inside a high-frequency induction heated furnace, where it gets heated by electric field coupling. The combustion reaction occurs under a stream of high-purity oxygen purge gas to ensure complete combustion. The carbonic and sulfuric species in the sample get oxidized by the oxygen flow and form carbon dioxide (CO_2_) and sulfur dioxide (SO_2_), respectively. The carrier gas passes through a dust filter and a drying reagent before passing through a set of non-dispersive infrared (NDIR) cells where carbon and sulfur content can be determined by measuring CO_2_ and SO_2_ contents in the analyte gas [40,41].

## 3. Characterization Results and Discussion

In this section the results of the characterized samples as well as challenges and insights from the different characterization methods used in this study are discussed.

### 3.1. Microstructural Analysis

**Figure 1:**
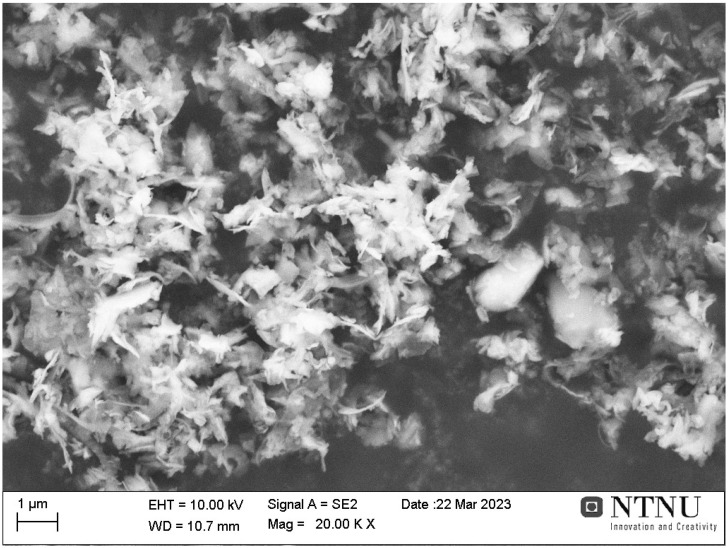
SEM micrograph of as-received kerf after drying.

The morphology of the as-received dried silicon kerf powder, analyzed by SEM, is shown in Figure 1.

**Figure 2:**
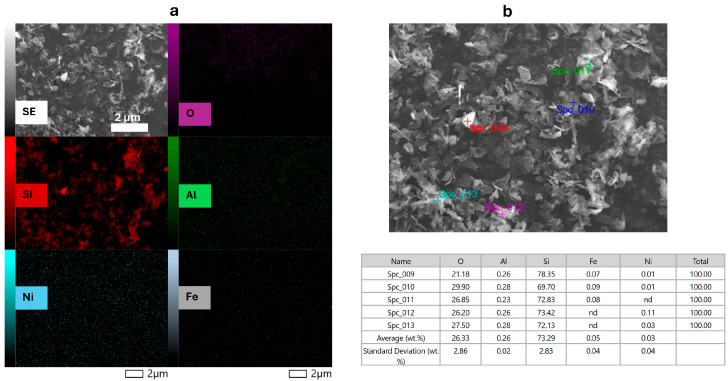
Elemental mapping by EDS (**a**) elemental distribution; (**b**) point analysis, of one of the samples investigated.

The image shows that the kerf particles are tiny flakes of loosely agglomerated material, irregular in shape and anisotropic with flake sizes ranging from several hundred nanometers to a few µm in length [42,43]. This morphology was uniform across all measured samples and agrees with current literature on silicon kerf samples. Element mapping by EDS shows the distribution of the major impurities on one selected sample (Figure 2a) as well as point analysis (Figure 2b) including some of the main metallic impurities (for the leaching treatment) of interest, i.e., Al, Ni and Fe.

### 3.2. Elemental Analysis

Key requirements in the recycling of kerf loss waste (KLW) are low content of boron (target < 0.08 ppmw), phosphorous (target < 0.3 ppmw) and metals (e.g., Al, Ca, Ni, Fe, etc.). As can be seen from Table 3 and Table 4, the impurities in KLW differ from source to source. However, the order of magnitude is quite comparable for most elements, showing that the general composition is quite similar across the different suppliers. One of the samples (REC5), however, was analyzed with 0.98 wt.% Al, which is quite different from the other 4 samples. The sample is also the oldest, and the high amount of Al can be attributed to the use of a slicing beam containing Al(OH)_3_ filler [43] while the other suppliers are likely to have used different beam material. The major metallic impurities in order of abundance are Ni, Al*, Ca, Fe and Mg.

Al* except for one sample with almost 1% Al.

Non-metallic impurities (C, O, N) were analyzed by the inert gas analysis (IGA) and infrared (IR)-combustion methods. The carbon content of the final purified Solar Grade Silicon is critical for the Czochralski (Cz) monocrystalline ingot pulling process. The acceptable C content in solar cell silicon is approximately < 5 ppmw, preferably lower. In KLW, the carbon content (mainly coming from the slicing beam) is in the percent range. Efficient carbon removal steps are thus a necessary part of a process to upgrade KLW to Solar Grade Silicon. The analyzed oxygen content is presumably mainly coming from surface SiO_2_ on the silicon particles and thus represents a yield loss. Oxygen analysis values as low as possible are desirable. Table 4 shows analysis for C, O and N. As can be seen in Table 4 the carbon percentage varies from ~1 to 4 wt.%, with an average of approximately 2 wt.% C. This can be attributed to different compositions of slicing beam and cutting fluids for different wafer manufacturers. The oxygen composition is quite consistent, around 4 wt.%, while the nitrogen composition is also very consistent, around 0.1 wt.%.

### 3.3. X-Ray Diffraction (XRD)

**Figure 3:**
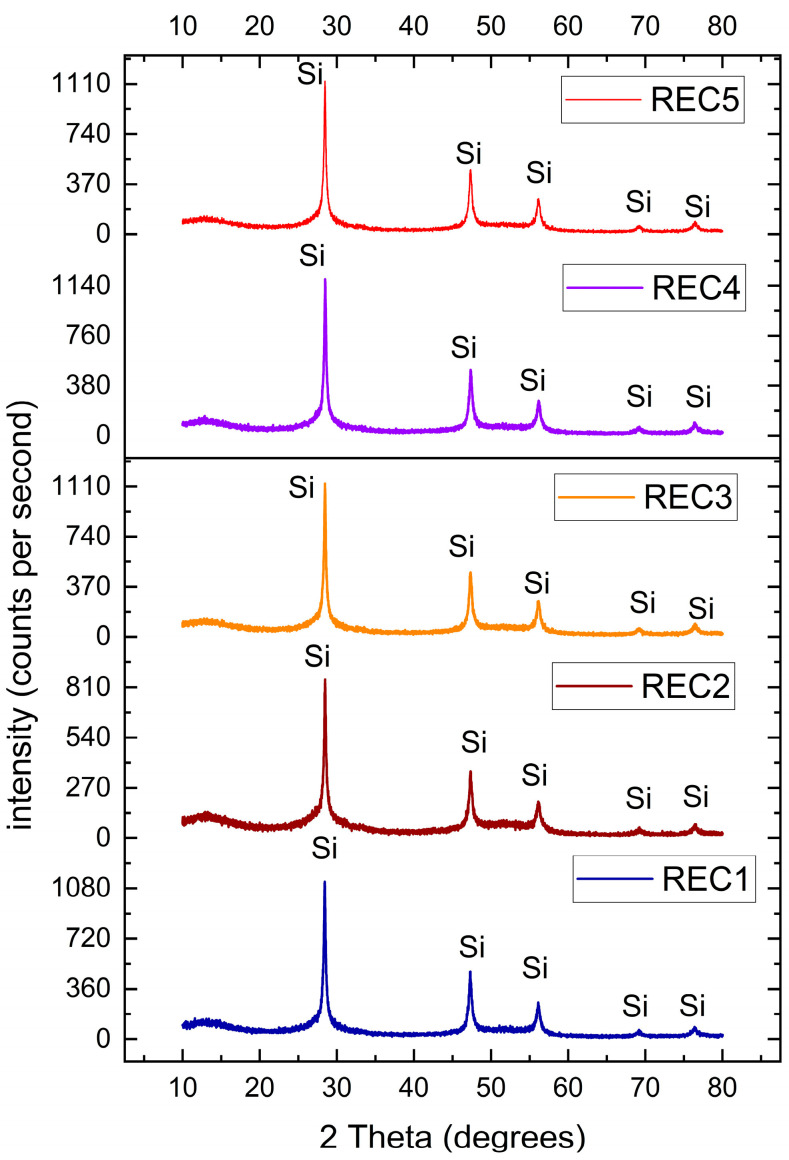
XRD diffractogram of powder kerf samples.

The XRD analysis in Figure 3 indicates single-phase crystalline Si in all the samples. The peaks occur at ~28.5°, 47.4°, 56.2°, 69.1° and 76.2°. The crystalline peaks are almost identical for all the five samples and are quite consistent with the literature on silicon kerf. This shows that the bulk of kerf loss waste is crystalline silicon.

The XRD patterns in Figure 3 also show that the silicon kerf samples contain small fractions of amorphous phases, which are amorphous silicon dioxide surface, and polymeric material particles. According to Table 4 the oxygen content of the Si-kerf samples is between 3.71 and 5.66 wt.%, and it may indicate that the quantity of amorphous SiO_2_ is two times greater than the oxygen content regarding the molecular weight of SiO_2_ (60 g/mol) with Si in it (28 g/mol). This means that surface amorphous SiO_2_ around the particles is in ranges of 7.95 to 12.1 wt.%.

### 3.4. Particle Size Distribution (PSD)

**Figure 4:**
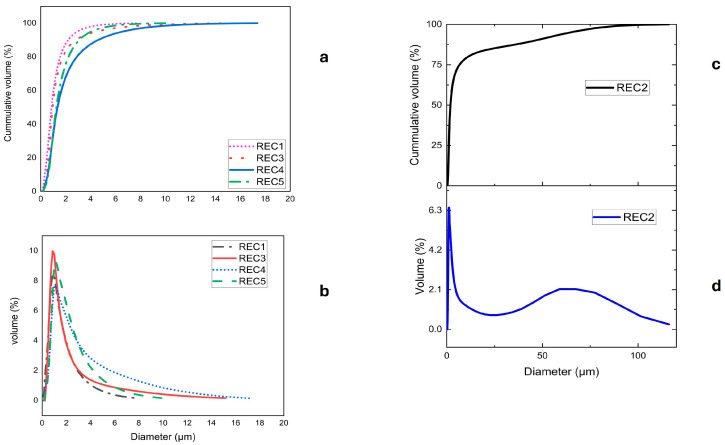
Particle size distribution for different samples: volume distribution for REC1,3,4&5 (**a**), cumulative volume distribution for REC1,3,4&5 (**b**), cumulative volume distribution for REC2 (**c**), and volume distribution for REC2, (**d**).

The particle size distribution of 4 of the 5 samples used showed a consistent particle size of 1–18 µm, with an average size (D50) of approximately 1–2 µm. However, one of the samples (REC2) showed a completely different particle size range, up to ~100 µm, and an average size of ~11 µm, as shown in Figure 4. Most of the samples have particle sizes within the expected range for silicon kerf. The difference in one of the samples could be a result of different cutting methods from one of the suppliers.

### 3.5. BET Surface Area

The surface area can give an indication of the quality of the cutting process during wafering. The results in Table 5 show a surface area range of ~30–40 m^2^/g for four of the five samples, whilst one sample (REC2) had close to half this value. This sample also behaved differently when particle size distribution was analyzed, showing differences in the cutting processes depending on supplier.

### 3.6. General Overview of Kerf Characteristics in the Analyzed Samples

In summary, the different samples of silicon kerf analyzed by different methods show quite similar trends across different characteristics. The morphology indicates that kerf loss waste is an inhomogeneous material made of irregular flakes of nano to micro scale as confirmed by SEM imaging. The main impurities are C (approximately 2 wt.%), O (approximately 4 wt.%) as well as metallic and doping impurities (approximately 400 ppmw in total). The silicon kerf powder of the analyzed samples was all crystalline as no amorphous peaks were detected. The average particle size is around 1–2 µm indicating that the kerf loss waste is composed of very fine particles. The average BET surface area is approximately 33 m^2^/g. The average concentration of dopant elements B, P and Ga is circa 0.08 ppmw, 1.6 ppmw and 1.4 ppmw respectively, which fall within the common PV level [43]. Table 6 summarizes the overall characteristics of the kerf samples analyzed in this study.

## 4. Characterization Methods and Techniques Discussion

### 4.1. GDMS: Peak Selection, Detection and Interferences

In GDMS, interference lines from residual gases or sputtered material can appear close to or even overlap with the peak of interest in the mass spectrum. The only element for which spectral overlap with atmospheric interference posed a significant issue was calcium. The primary isotope, ^40^Ca (96.94% abundancy), is completely obscured by ^40^Ar from the argon plasma. Therefore, the second most abundant isotope, ^44^Ca (2.086% abundancy), was used for quantification in this case. For all other elements, the most abundant isotopes were used for quantification.

**Figure 5:**
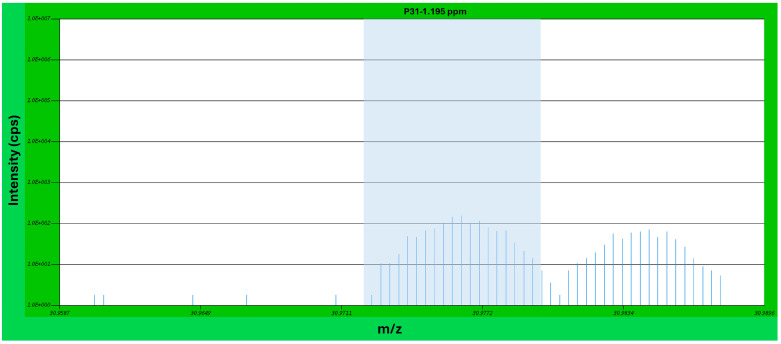
Mass spectrum of ^31^P, with a neighboring interference peak from a ^1^H^30^Si ion cluster.

In many cases, additional peaks were observed adjacent to the target analyte peaks. However, in all such cases, operating at a mass resolution of 4000 (M/ΔM, defined at 10% of peak height) provided sufficient peak separation to ensure accurate quantification. Figure 5 shows an example mass spectrum of ^31^P, with a neighboring but well-separated interference peak from a ^1^H^30^Si ion cluster to the right of the ^31^P peak. Additional examples of such plots are provided in the Supplementary Materials.

Table 7 lists several elements that exhibit interferences or adjacent peaks, which may affect quantification when measurements are performed at a mass resolution below 4000.

The intensity of atmospheric interference peaks varies depending on the sample and the vacuum level in the glow discharge chamber. These signals typically decrease significantly during the pre-sputtering phase.

#### Solid vs. Powder Samples: Insights

When inserting a powder Si sample pressed into an indium mask, the discharge voltage and current parameters typically take somewhat longer to stabilize compared to solid silicon samples. Matrix signal levels are usually about one order of magnitude lower in powder samples than in solid Si, for example, in the 10^−10^ A range for solid samples versus the 10^−11^ A range for Si powders. This reduction is due to the smaller amount of analyte material available in powder samples. Because of the lower signal intensity, longer integration times for trace elements may be required to improve counting statistics. In practice, however, this has not posed a problem for quantification. As the analysis of powder samples is prone to inhomogeneity, each sample measurement was performed with 10 repetitions until a steady-state signal was achieved and low RSDs were obtained.

### 4.2. SIMS: Peak Selection, Detection and Interferences

**Figure 6:**
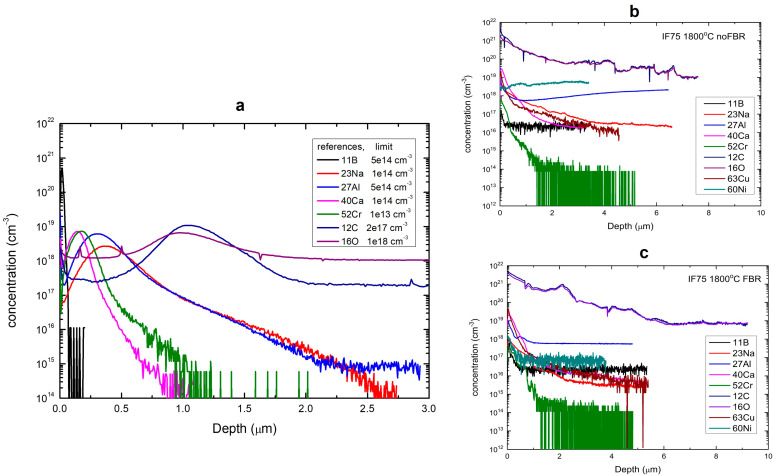
Concentration vs. depth profiles of different elements as measured by SIMS in (**a**) the ion implanted samples used for calibration and (**b**,**c**) in the two different samples as indicated in the corresponding legends. The detection limits of different elements are shown in panel (**a**).

For SIMS analysis, two solid Si samples (recycled from Si-kerf) were analyzed, as shown in Figure 6. Specifically, Figure 6a shows the concentration vs. depth profiles of different impurities in the reference ion implanted samples which were used for calibration. It should be noted that the 15 keV Cs analyzing beam was used for the detection of C and O, while 10 keV O_2_ ions were used for the rest of the measured elements. The concentration detection limits indicated in the legend in panel (a) were determined as a minimum concentration behind the implanted peaks. Figure 6b,c show the SIMS results for the two different samples processed at 1800 °C (c) with and (b) without FBR.

It is seen that there is an enhanced concentration of all the elements near the surface region that might be attributed to the segregation of impurities during solidification. However, the concentrations exhibit a lowering trend with increasing depth, and they become stable for the depth more than 4 μm. These bulk concentration values were indicated in Table 8 summarizing the impurity values in the measured samples alongside the concentration detection limits.

### 4.3. Inert Gas Analysis (IGA) and Infrared (IR)-Combustion Method: Analysis and Insights

The main challenge when analyzing silicon kerf samples with inert gas analysis (IGA) and infrared (IR)-combustion method is the inhomogeneity of the material. This means that there are potentially large variations in the elemental compositions within the sample, which gives rise to large uncertainties and high relative standard deviations (RSD). Table 9 lists the carbon and oxygen values measured with the LECO instruments for a series of different types of silicon kerf samples. Samples measured after froth flotation treatment, with much finer particles (after deagglomeration before flotation), are referred to as ‘feed’ and ‘tails’, while those analyzed after thermal decarburization, with coarser particles (as there was no deagglomeration of the kerf cake), are referred to as ‘decarburized’ samples.

Analysis of feed and tail samples results in relatively high carbon concentrations with low RSD values, while for the decarburized samples the RSD values are much more variable and significantly higher. For silicon kerf to be recycled back to a usable product for PV solar applications, the impurity content must be controlled. Decarburization via heat treatment of silicon kerf successfully lowers the carbon content compared to the original sample and feed/tail samples in general, as evidenced from the results listed in Table 9. This is great news for the application of the kerf product, but it brings some challenges when it comes to the analysis of the elemental composition. Samples with low elemental concentration are even more susceptible to higher uncertainties and RSD, which is also the case for the listed samples. This is also true for low O-content samples, for instance for PV solar Si samples. For the decarburized samples, the oxygen content is rather unaffected but also suffers from large uncertainties as a result of inhomogeneity.

There are several steps that can be taken to help improve the issue of inhomogeneity. When analyzing samples with the LECO apparatuses, only a small sample amount is used. This means that it is very important that the fraction that you select is representative of the entire sample that you want to analyze. Meticulous sample preparation is therefore important.

For optimal accuracy, it is recommended that the sample is ground to a fine and uniformly mixed powder. If larger sample fractions are to be analyzed, it is important to run parallels from different parts of the bulk sample to achieve representative sampling. Furthermore, when handling samples with low oxygen or carbon contents, increasing the sample mass will increase the amount of oxygen or carbon in the analyzed sample and give increased signal strength. Increasing sample mass can also be helpful for heterogeneous samples as it gives a more representative sample batch. However, there are some limitations to what the instrument is capable of.

It is also crucial that no contaminations are introduced, especially relating to the desired element to be analyzed. Even small impurities will influence the analytical results, particularly in samples with low oxygen or carbon contents. This goes for both the sample itself and the crucible material used during the combustion reactions, particularly during C-determination using the LECO CS844 instrument. Here, ceramic crucibles are used, which could introduce variability if not handled correctly. They should not be touched with bare hands, only with clean tools. ASTM and ISO standards also call for preheating the crucibles in a muffle furnace at 1000 °C for 2 h or above 1250 °C for at least 15 min. This is especially important for low carbon-containing samples but would also be relevant for higher C-contents where the sampling mass is low. For the graphite crucibles used in LECO ON836, high-purity graphite material is used, and no further pre-treatment is necessary. Furthermore, the crucibles get outgassed multiple times in the instrument before the sample gets added, which reduces contaminations.

In addition to the more practical sample handling measures described above, there are also other means to combat the challenges with inhomogeneity. An important aspect is to run additional parallels for each sample. Commonly, three parallels for each sample are sufficient to give a good representative average, given that the uncertainty is low enough. For particularly heterogeneous samples, running more parallels will improve confidence and reduce sampling errors.

## 5. Conclusions

Five industrial silicon kerf samples from different suppliers have been characterized, and similarities and differences have been evaluated. The following conclusions were drawn:

1. The surface morphology, according to SEM measurements, indicated that silicon kerf is an inhomogeneous material made up of irregular nano- and micro-scale flakes.
2. The average metallic impurity concentration (for four of the five samples) was about 400 ppmw; O and C concentrations were approximately 4.6 and 2.3 wt.%, respectively.
3. The average particle size (D50) was about 3.5 µm for the five samples, while the average BET surface area was approximately 33 m^2^/g.
4. Analysis of powder kerf samples showed possible interferences between several isotopes. However, operating at a mass resolution of 4000 (M/ΔM, defined at 10% peak height) provided sufficient peak separation.
5. The inhomogeneity of kerf samples can present challenges in analyzing them by inert gas analysis (IGA) and infrared (IR)-combustion methods and samples with low elemental concentration were found to be more susceptible to higher uncertainties and RSD.

Based on these results, we would recommend that when analyzing silicon kerf (saw dust) it is important to do meticulous sampling, running several parallels, and maybe testing each batch individually before mixing (for large scale) operations.

## Figures and Tables

**Table 1:** Measured and specified element concentrations of 7N indium masks provided by RASA Industries.

Element	Actual (ppmw)	Specification (ppmw)
Boron	<0.001	—
Magnesium	<0.001	≤0.01
Aluminum	<0.005	≤0.03
Silicon	<0.005	≤0.03
Phosphorus	<0.001	—
Calcium	<0.01	≤0.03
Titanium	<0.001	≤0.01
Vanadium	<0.001	<0.009
Chromium	<0.002	<0.01
Manganese	<0.001	<0.01
Iron	<0.004	≤0.03
Nickel	<0.002	<0.02
Copper	<0.003	<0.02
Gallium	<0.006	—

**Table 2:** Instrument ranges and precisions for the two LECO tools, ON836 and CS844, used in this study [37]. * Reducing the sample size shifts the range, i.e., 0.1 g of sample gives a O-range of 0.5 ppm–50% or a C-range of 6 ppm–60%.

	ON836		CS844	
	Oxygen	Nitrogen	Carbon	Sulfur
Instrument range * (1 g sample)	0.00005 mg–50 mg (0.05 ppm–5%)	0.00005 mg–30 mg (0.05 ppm–3%)	0.0006 mg–60 mg (0.6 ppm–6%)	0.0006 mg–60 mg (0.6 ppm–6%)
Instrument precision	0.000025 mg or 0.3% RSD, whichever is greatest	0.000025 mg or 0.3% RSD, whichever is greatest	0.0003 mg or 0.5% RSD, whichever is greatest	0.0003 mg or 0.5% RSD, whichever is greatest

**Table 3:** Impurity content from ICP-OES/MS (ppmw).

	REC1	REC2	REC3	REC4	REC5
Al	6.3	126.6	165.5	114.3	9800
B	0.02	0.02	0.03	0.09	0.25
Ca	84.2	44.7	53.0	22.4	14.6
Cr	<0.7	<0.7	1.0	<0.7	<0.7
Cu	<0.5	<0.5	<0.5	<0.5	0.7
Fe	6.5	25.1	50.3	18.3	2.5
Ga	0.9	1.5	1.7	1.5	2.6
Mg	27	2.1	16.4	1.6	2.7
Mn	<0.4	<0.4	0.4	0.5	<0.4
Ni	236	246	83.8	257	114
P	<0.6	1.01	3.85	0.9	1.02
Ti	<0.4	1.1	3.4	1.0	3.4
V	<0.4	<0.4	<0.4	<0.4	<0.4

**Table 4:** C, O and N composition from IGA and IR-combustion.

Sample	Carbon (wt.%)	Oxygen (wt.%)	Nitrogen (wt.%)
REC1	2.62	4.65	0.13
REC2	4.16	4.47	0.14
REC3	2.42	4.36	0.12
REC4	1.39	3.71	0.13
REC5	0.951	5.66	0.11

**Table 5:** The measured BET surface area of the kerf samples.

Sample	BET Surface Area (m^2^/g)
REC1	39.8
REC2	17.7
REC3	41.7
REC4	35.7
REC5	30.4

**Table 6:** Summary of the overall characteristics of silicon kerf samples.

Surface morphology	Inhomogeneous, irregular nano- to micro-scale flakes.
Crystallinity	Crystalline Si peaks
Average metallic impurity conc. (ppmw)	~ 400 (excluding high Al sample), ~10 000 including high Al sample.
Average O conc. (wt.%)	~4.6
Average C conc. (wt.%)	~2.3
Average BET surface area (m^2^/g)	~33
Average particle size (D50) (µm)	~3.5 (including sample 2), ~1.6 (excluding sample 2)
Average P conc. (ppmw)	~1.6
Average B conc. (ppmw)	~0.08
Average Ga conc. (ppmw)	~1.4

**Table 7:** Isotopes with potential interferences (from ref. [44] and Nu Astrum Data Analysis V1.2.6387.13884).

Isotope	Potential Interference(s)
^31^P	^1^H^30^Si
^48^Ti	^12^C^36^Ar
^52^Cr	^12^C^40^Ar
^56^Fe	^16^O^40^Ar and/or ^28^Si^28^Si
^55^Mn	^17^O^38^Ar
^58^Ni	^28^Si^30^Si

**Table 8:** Summary of the analyzed solid Si samples as well as the individual elemental detection limits.

Sample	^11^B	^23^Na	^27^Al	^40^Ca	^52^Cr	^12^C	^16^O	^63^Cu	^60^Ni
FBR	2 × 10^16^	2 × 10^15^	6 × 10^17^	4 × 10^15^	<1 × 10^13^	7 × 10^18^	7 × 10^18^	<1 × 10^15^	1 × 10^17^
no FBR	2 × 10^16^	2 × 10^16^	2 × 10^18^	2 × 10^16^	<1 × 10^13^	1 × 10^19^	1 × 10^19^	7 × 10^15^	5 × 10^18^
detection limit	5 × 10^14^	1 × 10^14^	5 × 10^14^	1 × 10^14^	1 × 10^13^	2 × 10^17^	1 × 10^18^	1 × 10^15^	1 × 10^17^

**Table 9:** A selection of carbon and oxygen contents of various types of silicon kerf samples, with uncertainties and relative standard deviation (RSD).

Sample Type	Carbon (wt.%)	RSD, Carbon (%)	Oxygen (wt.%)	RSD, Oxygen (%)
Feed	1.94 ± 0.03	1.55	-	-
	1.59 ± 0.06	3.77	-	-
Tails	2.42 ± 0.02	0.83	-	-
	1.99 ± 0.04	2.01	-	-
Original sample before decarburization	3.28 ± 0.12	3.66	5.97 ± 1.35	22.61
Decarburized samples	0.0595 ± 0.0161	27.06	7.60 ± 0.33	4.34
	0.0538 ± 0.0032	5.95	9.01 ± 0.35	3.88
	0.0681 ± 0.0038	5.58	13.30 ± 2.33	17.52
	0.1122 ± 0.0193	17.20	5.74 ± 0.24	4.18
	0.0558 ± 0.0048	8.60	6.94 ± 0.23	3.31
	0.2128 ± 0.0248	11.65	5.54 ± 0.30	5.42
	0.0518 ± 0.0138	26.64	10.40 ± 1.73	16.63
	0.0492 ± 0.0035	7.11	9.20 ± 0.65	7.07

## Data Availability

The original contributions presented in this study are included in the article/supplementary material. Further inquiries can be directed to the corresponding author.

## Supplementary Materials

The following supporting information can be downloaded at: https://www.mdpi.com/article/10.3390/ma19030541/s1, Figure S1: Top-Mass spectrum of 44Ca and 16O28Si ion cluster peak, Bottom-Mass spectrum of 58Ti and 12C36Ar ion cluster peak; Figure S2: Top-Mass spectrum of 52Cr and 12C40Ar ion cluster peaks, Bottom-Magnet scan window with 56Fe and 16O40Ar and/or 28Si28Si ion cluster peaks; Figure S3: Top-Magnet scan window with 55Mn and 17O38Ar ion cluster peak, Bottom-Magnet scan window with 58Ni and 28Si30Si ion cluster peak.
