# Supplementary material for: Challenges and Practices in the Analysis of Silicon Kerf from the PV Industry by Combinatorial Analytical Methods
Source: Materials (Basel). 2026 Jan 29;19(3):541. doi: 10.3390/ma19030541 (PMC12897825; doi:10.3390/ma19030541)
Supplement: Supplementary file 1 [file materials-19-00541-s001.zip › materials-4085854-supplementary.pdf]

materials

MDPI

Supplementary Materials

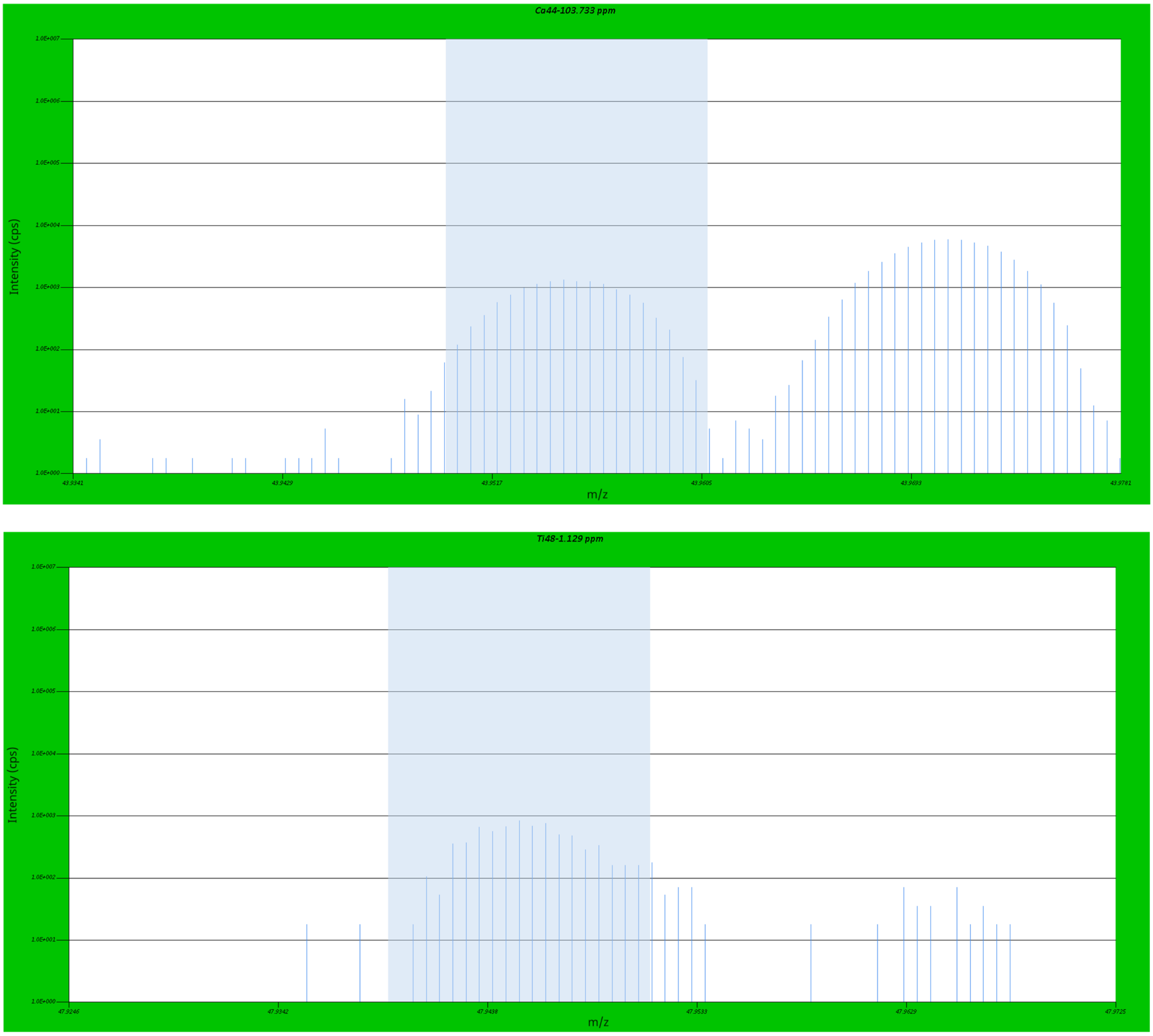

Figure S1. Top-Mass spectrum of 44Ca and 16O28Si ion cluster peak, Bottom-Mass spectrum of 58Ti and 12C36Ar ion cluster peak.

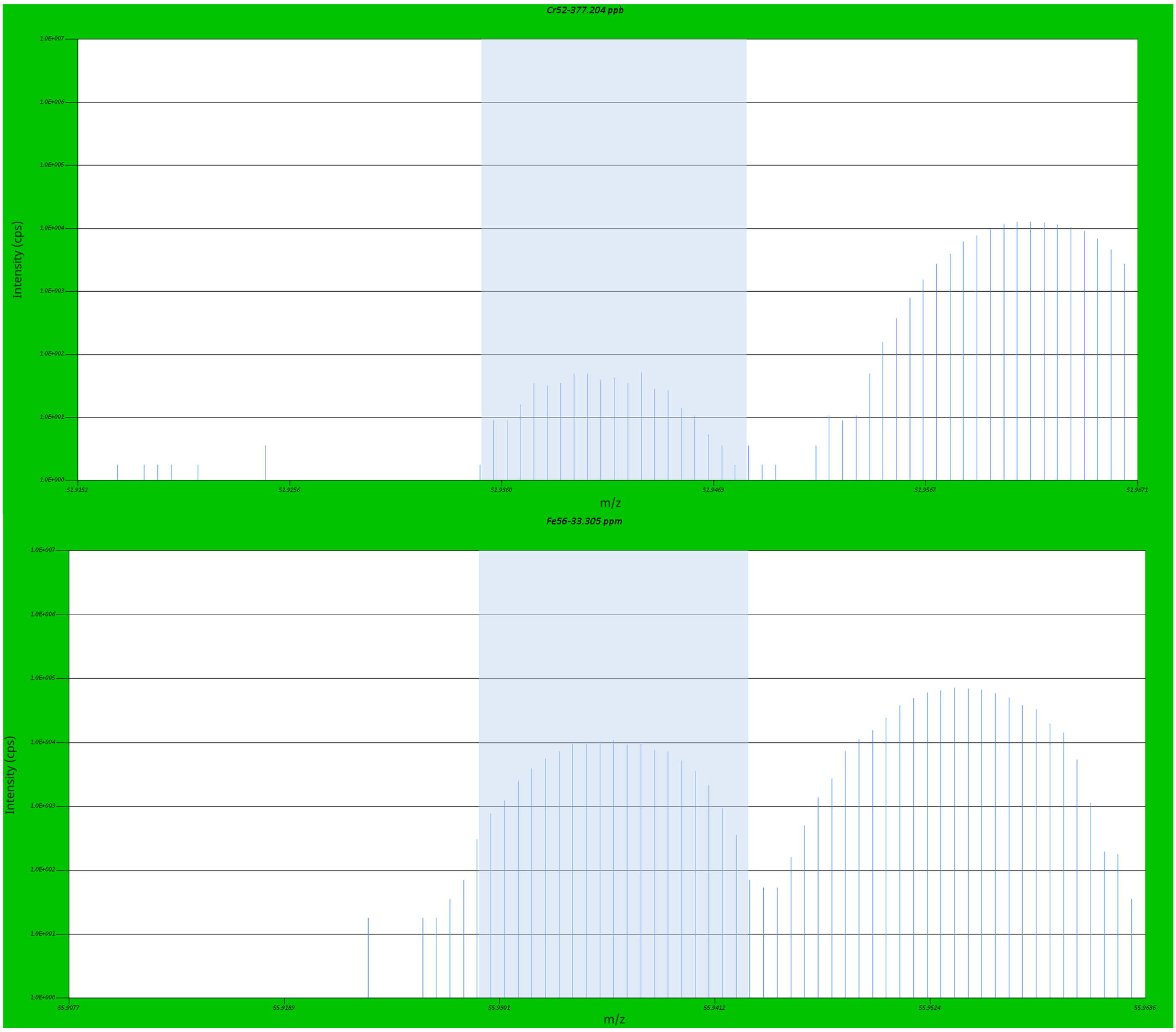

Figure S2. Top-Mass spectrum of ^{52}Cr and $^{12}C^{40}Ar$ ion cluster peaks, Bottom-Magnet scan window with ^{56}Fe and $^{16}O^{40}Ar$ and/or $^{28}Si^{28}Si$ ion cluster peaks.

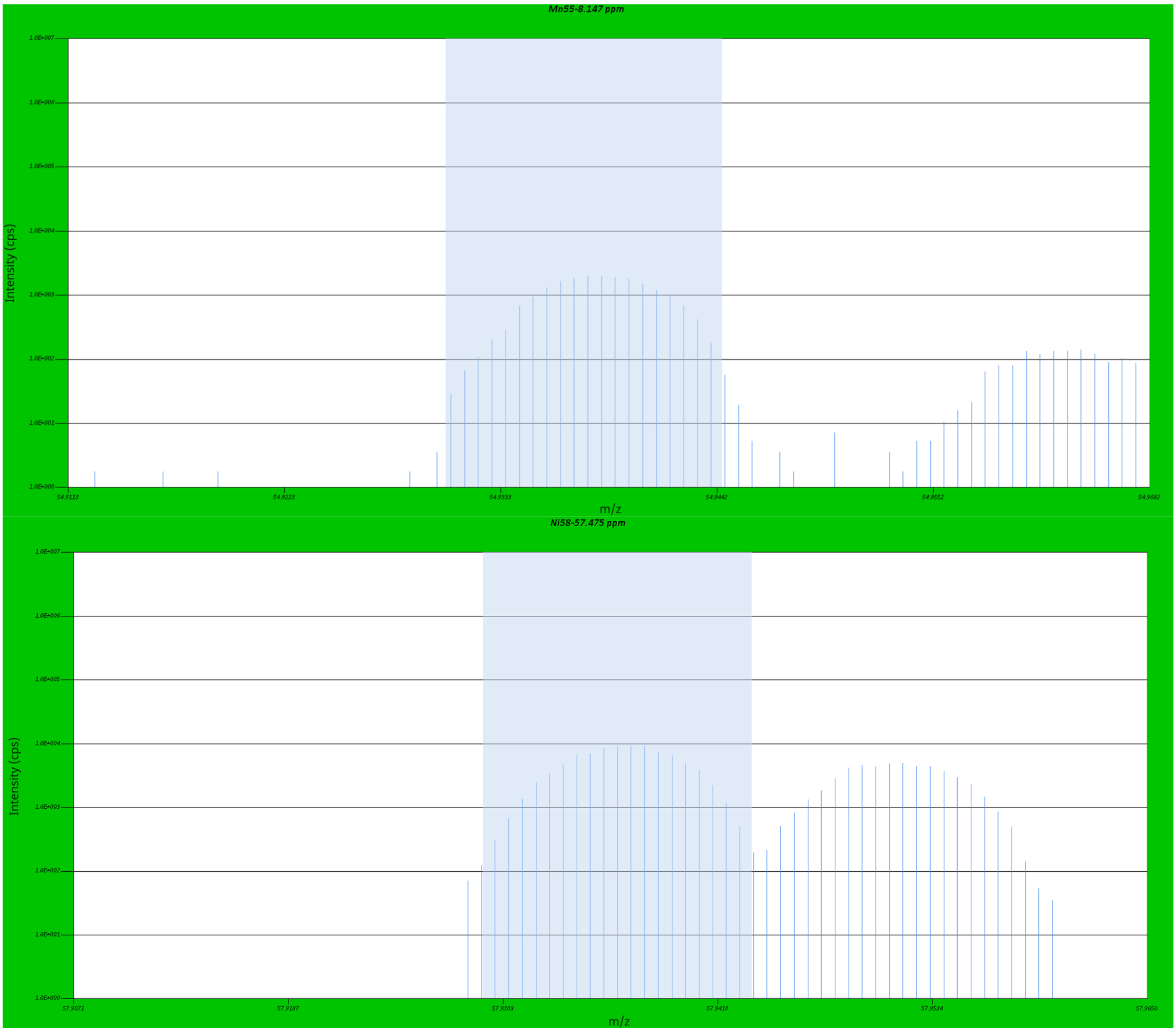

Figure S3. Top-Magnet scan window with ^{55}Mn and $^{17}O^{38}Ar$ ion cluster peak, Bottom-Magnet scan window with ^{58}Ni and $^{28}Si^{30}Si$ ion cluster peak.
